# The *OsCYP19-4* Gene Is Expressed as Multiple Alternatively Spliced Transcripts Encoding Isoforms with Distinct Cellular Localizations and PPIase Activities under Cold Stress

**DOI:** 10.3390/ijms17071154

**Published:** 2016-07-19

**Authors:** Areum Lee, Sang Sook Lee, Won Yong Jung, Hyun Ji Park, Bo Ra Lim, Hyun-Soon Kim, Jun Cheul Ahn, Hye Sun Cho

**Affiliations:** 1 Biosystems and Bioengineering Program, University of Science and Technology (UST), Daejeon 34113, Korea; lar1027@kribb.re.kr; 2 Molecular Biofarming Research Center, Korea Research Institute of Bioscience and Biotechnology, Daejeon 34141, Korea; sosimsimgo2016@gmail.com (S.S.L.); jwy95@kribb.re.kr (W.Y.J.); hjpark@kribb.re.kr (H.J.P.); lbr217@kribb.re.kr (B.R.L.); hyuns@kribb.re.kr (H.-S.K.); 3 Department of Pharmacology, College of Medicine, Seonam University, Namwon 55724, Korea; secmeta2@seonam.ac.kr

**Keywords:** alternative splicing, cold stress, isoform, OsCYP19-4, PPIase activity, RCN1 interactor

## Abstract

Alternative splicing (AS) is an important molecular mechanism by which single genes can generate multiple mRNA isoforms. We reported previously that, in *Oryza sativa*, the *cyclophilin 19-4* (*OsCYP19-4.1*) transcript was significantly upregulated in response to cold stress, and that transgenic plants were cold tolerant. Here we show that, under cold stress, *OsCYP19-4* produces eight transcript variants by intron retention and exon skipping, resulting in production of four distinct protein isoforms. The OsCYP19-4 AS isoforms exhibited different cellular localizations in the epidermal cells: in contrast to OsCYP19-4.1, the OsCYP19-4.2 and OsCYP19-4.3 proteins were primarily targeted to guard and subsidiary cells, whereas OsCYP19-4.5, which consists largely of an endoplasmic reticulum (ER) targeting signal, was co-localized with the RFP-BiP marker in the ER. In OsCYP19-4.2, the key residues of the PPIase domain are altered; consistent with this, recombinant OsCYP19-4.2 had significantly lower PPIase activity than OsCYP19-4.1 in vitro. Specific protein-protein interactions between OsCYP19-4.2/3 and AtRCN1 were verified in yeast two-hybrid (Y2H) and bimolecular fluoresence complementation (BiFC assays), although the OsCYP19-4 isoforms could not bind each other. Based on these results, we propose that two OsCYP19-4 AS isoforms, OsCYP19-4.2 and OsCYP19-4.3, play roles linking auxin transport and cold stress via interactions with RCN1.

## 1. Introduction

Alternative splicing (AS) is a fundamental process of gene regulation and depends on differential splice site selection, which is regulated by exonic and intronic sequences called splicing enhancers or suppressor elements that yield multiple mRNA transcripts from the same pre-mRNA [1]. The spliced variants can encode proteins with different functions, thereby increasing functional diversity [2]. However, some AS events produce variants that contain a premature termination codon (PTC); these mRNAs are targeted for RNA degradation in the cytoplasm via nonsense-mediated decay (NMD). Thus PTCs affect transcript levels or give rise to truncated proteins [3]. AS is a common co- or post-transcriptional process in eukaryotes. Recent genome level analyses revealed that more than 95% of human genes with multiple exons undergo AS [4], whereas up to 60% and 48% of intron-containing genes are alternatively spliced in the model plants *Arabidopsis thaliana* [5] and rice [6], respectively. Recent plant studies revealed that AS can be regulated by growth and developmental stage, the circadian clock, and responses to abiotic and biotic stress [7].

Cold stress has a major impact on plant growth and development, significantly constraining the geographical distribution of plant species as well as agricultural quality and productivity [8]. For adaption to low temperature, plants alter their physiological and biochemical processes via transcriptional, post-transcriptional and post-translational mechanisms [9,10]. Genome-wide transcriptome studies showed that 10%‒45% of *Arabidopsis* transcripts are regulated during acclimation to low temperature [11,12]. Cold stress also significantly induces AS in plants; consistent with this, the expression and splicing of many of serine/arginine-rich (SR) genes, which are essential for constitutive and alternative splicing, are regulated by cold stress [13]. In response to cold stress, AS genes play important roles in multiple signal transduction pathways, including starch metabolism [14], circadian clock [15], and light and stress signaling [16]. A recent tiling array study in *Arabidopsis* showed that the majority of cold-regulated AS events result in PTC-containing transcripts, and revealed new types of cold-responsive genes [17].

In response to low temperature, plants adopt various strategies to increase their cold tolerance. In a previous study in *Oryza sativa*, we showed that *cyclophilin 19-4* (*OsCYP19-4*) is involved in cold acclimation and characterized this gene in the context of developing cold tolerant crops [18]. Here, we report that, under low temperature conditions, the primary *OsCYP19-4* transcript generated eight alternative splice forms via two types of regulated splicing events, intron retention and exon skipping. We examined the subcellular localization of these OsCYP19-4 isoforms in plant cells using GFP-fusion constructs. In addition, using yeast two hybrid (Y2H) and bimolecular fluorescence complementation (BiFC) assays, we detected specific protein-protein interactions between the OsCYP19-4.2/3 AS isoforms and a regulatory α-subunit of PP2A (AtRCN1). Despite their lack of PPIase activity, these two isoforms were specifically localized to guard or subsidiary cells. Taken together, our data indicate that *OsCYP19-4* is multiply alternatively spliced and that most of the splice variants encode immunophilins with nonfunctional PPIase domains. Via their interactions with RCN1, AS isoforms play regulatory roles that link auxin transport to cold stress. These findings provide insights into the functions of spliced isoforms and the fine-tuning of gene regulation in response to cold stress.

## 2. Results

### 2.1. OsCYP19-4 Produces Extensive AS Isoforms in a Cold-Specific Manner

In a previous study, we showed that *OsCYP19-4* expression increased dramatically in response to cold stress. Subsequent experiments revealed that, under these conditions, the *OsCYP19-4* gene produces multiple RT-PCR fragments from full-length *OsCYP19-4* mRNA, indicating the production of multiple isoforms by AS. *OsCYP19-4* expression increased gradually during cold stress, and multiple AS variants were concurrently observed until 48 h after cold treatment (Supplementary Figure S1A). RT-PCR detected eight transcript variants of *OsCYP19-4* under cold stress, the main band, one smaller band, and six larger bands, using one primer encompassing the ATG codon and another encompassing the stop codon for *OsCYP19-4* (Supplementary Figure S1B). To confirm the absence of genomic DNA contamination, we performed RT-PCR analysis under the same conditions without the reverse transcriptase enzyme (Supplementary Figure S1C).

### 2.2. Gene Structures of Alternatively Spliced OsCYP19-4 Transcripts

**Figure 1:**
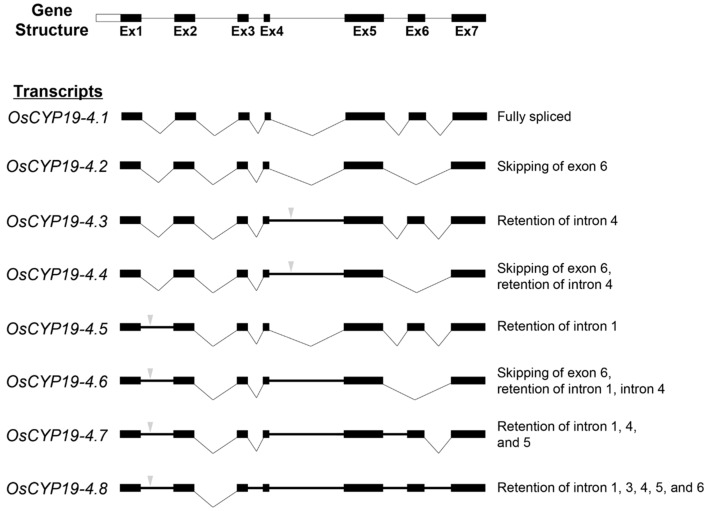
Schematic diagram illustrating the alternative splicing events of *OsCYP19-4* during cold stress. Gene structure of *OsCYP19-4*. Scheme of the fully spliced mRNA and seven alternative splicing (AS) isoforms of *OsCYP19-4*. Black boxes represent the seven exons, whereas the six intronic regions are shown as straight or bent (“V”) lines. Bold lines represent intron retentions. Inverted triangle (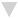) indicates premature termination codons (PTCs). AS types are represented at the right.

To confirm that these PCR products arose from *OsCYP19-4* and represented novel AS events, we mapped their nucleotide sequences onto the genomic structure of this gene. *OsCYP19-4* consists of seven exons and six introns, yielding a 730-nucleotide transcript under normal conditions (*OsCYP19-4.1*). AS resulted in significant changes over a wide range of sequences, including skipping of exon 6 (*OsCYP19-4.2*) or retention of introns 1, 3, 4, 5 and 6 (*OsCYP19-4.3-8*). Retention of intron 1 or 4 introduces a PTC in *OsCYP19-4.3-8*, resulting in either production of truncated proteins or NMD (Supplementary Figure S2). These PTCs may affect the stability and/or translatability of the mRNAs. The second intron was the only *OsCYP19-4* intron whose splicing was not affected following exposure to cold stress. The predicted structures of *OsCYP19-4* AS transcripts and AS types are represented in Figure 1.

### 2.3. Analysis of the 5′and 3′ Splice Sites in All Introns of OsCYP19-4

**Figure 2:**
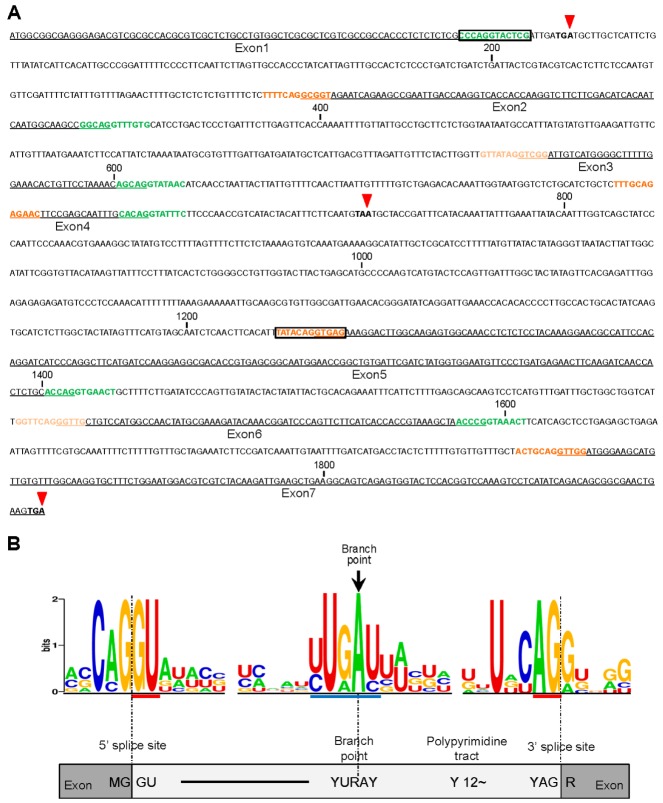
Nucleotide sequence annotation and splice site prediction in *OsCYP19-4* DNA. (**A**) The positions of splice sites in *OsCYP19-4.* Exon sequences are underlined, and constitutive 5′ and 3′ splice sites (predicted by ASSP, NetGene2, and SplicePort tools) are indicated in green and orange, respectively. Boxes represent putative dual-aspect splice sites identified using the ASSP tool. PTCs are indicated by inverted red arrows. Numbers indicate base pairs; (**B**) Consensus sequences obtained from splice site prediction in *OsCYP19-4*. Consensus sequences of 5′ and 3′ splice sites and branch points were derived from multiple sequence alignment of six introns in *OsCYP19-4* and represented as annotated sequences using WebLogo. Blue and red bars indicate the boundaries of each functional sequence. The frequencies of A, C, G and T at each position are represented by the relative height of the corresponding letter. Schematic diagram below shows two exons and an intron. R = A or G; Y = C or T.

To determine if variation in the splice site core sequence correlates with the frequency of splicing taking place at this sequence, we predicted the splicing sites from the *OsCYP19-4* genomic DNA sequence using the Aletrnative Splice Site Predictor (ASSP) [19], NetGene2 [20] and SplicePort [21] programs. The results revealed six (green) and six (red) consensus sequences as 5′ and 3′ splice sites, respectively (Figure 2). In addition, we found two dual-aspect splice sites (donor and acceptor functions are both possible); one in the 3′ splice site of intron 1 (positions 81 and 82 were predicted donor and acceptor site, respectively) and the other in the 5′ splice site of intron 4 (positions 1223 and 1224 were predicted donor and acceptor site, respectively) using the ASSP tool (Figure 2A, boxed). These sites potentially lead to AS. The identified features of the intronic 5′ and 3′ splice sites were grouped into clusters by MEME, and the frequency plot sequence logos were generated for each cluster using the WebLogo tool. The positions of 5′ and 3′ splice sites (GU and AG dinucleotides, respectively) and branch point sites (YURAY) of intron‒exon splice boundaries in the *OsCYP19-4* gene have the same core sequences without any change in the sequences (Figure 2B). This result indicates that intronic splice sites of *OsCYP19-4* are highly conserved as consensus sequences, despite the fact that it is multiply alternatively spliced.

### 2.4. Biochemical Properties of Proteins Encoded by Alternatively Spliced Isoforms of OsCYP19-4

**Figure 3:**
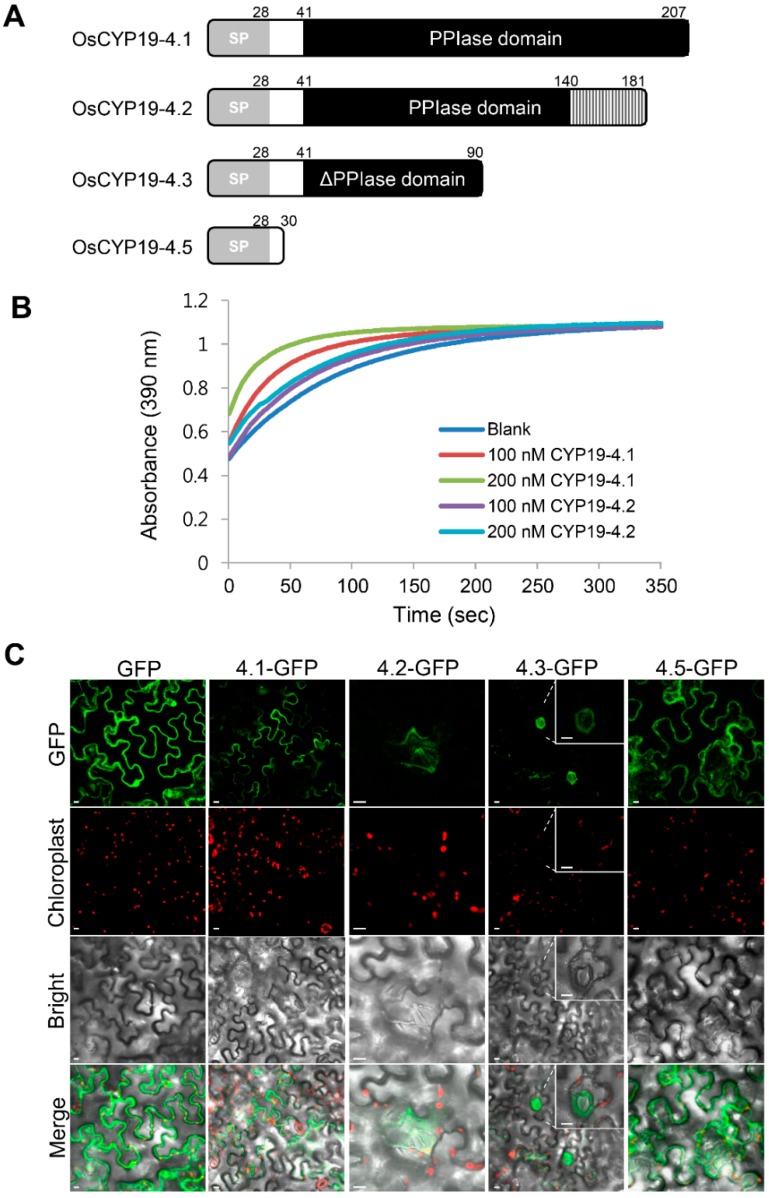
Characteristics of OsCYP19-4 AS isoform proteins. (**A**) Domain structures of AS isoform proteins. SP, signal peptides; PPIase domain, peptidyl prolyl *cis/trans* isomerase domain; ΔPPIase domain, deleted peptidyl prolyl cis/trans isomerase domain. Numbers indicate amino acids; (**B**) PPIase activity of recombinant OsCYP19-4.2 AS protein. Curves represent isomerization of the Suc-AAPF-pNA substrate at 10 °C over the course of 300 s in the absence of OsCYP19-4 (Blank; blue) and in the presence of 100 nM (OsCYP19-4.1: red; OsCYP19-4.2: purple) or 200 nM (OsCYP19-4.1: green; OsCYP19-4.2: sky blue) recombinant proteins; absorbance was measured at 390 nm; (**C**) Subcellular localization of GFP-fused fully spliced and AS OsCYP19-4 proteins. GFP fluorescence and chlorophyll autofluorescence are shown in green and red color, respectively. Merged images show GFP and chloroplast fluorescence. Bright field images show epidermal cells of *Nicotiana benthamiana* infected with *Agrobacterium* GV3101 harboring OsCYP19-4.1-GFP (4.1-GFP), OsCYP19-4.2-GFP (4.2-GFP), OsCYP19-4.3-GFP (4.3-GFP), or OsCYP19-4.5-GFP (4.5-GFP). Scale bars = 10 μm.

*OsCYP19-4* generates eight AS isoforms, of which six contain PTCs. These splice variants are predicted to encode proteins with altered structural organization. A ClustalW alignment of the amino acid sequences of the proteins encoded by OsCYP19-4 AS isoforms is displayed in Supplementary Figure S2; amino acids that are identical or similar are shaded in black or gray, respectively. AS of *OsCYP19-4* produces three alternative protein isoforms resulting from intron retention and exon skipping. Among the AS isoforms, only OsCYP19-4.2 encompasses the PPIase domain. Skipping of exon 6 in the *OsCYP19-4.2* transcript yields a protein 26 aa shorter than OsCYP19-4 and 6 of the 14 residues crucial for PPIase activity are altered, suggesting that the PPIase activities of OsCYP19-4.1 and OsCYP19-4.2 would be different (Supplementary Figure S2). The OsCYP19-4.3/4 AS transcripts encode identical proteins, but a PTC in retained intron 4 results in the production of a C-terminally truncated protein lacking all key residues for PPIase activity; therefore, these isoforms are likely to have impaired function. OsCYP19-4.5/6/7/8 AS transcripts also encode identical proteins, but due to a PTC in retained intron 1, the proteins are only 30 aa long and consist largely of the ER signal sequence: therefore, they are probably nonfunctional (Figure 3A).

To measure the PPIase activity of OsCYP19-4.2, we expressed recombinant His-tagged OsCYP19-4.2 in *Escherichia coli* (Supplementary Figure S3). Compared to OsCYP19-4.1-His, OsCYP19-4.2-His had greatly reduced PPIase activity, almost identical to the level in the blank control (Figure 3B). Thus, the OsCYP19-4.2 protein had an inadequate PPIase domain, and consequently had negligible PPIase activity in vitro.

To determine whether OsCYP19-4 AS isoforms have different functions, we investigated their localizations in plant cells. For this purpose, OsCYP19-4 AS isoforms were individually cloned into the plant expression vector pCAMBIA1302 between the 35S promoter and GFP, and their subcellular locations were characterized by transient expression of the target gene and GFP in *N. benthamiana* epidermal cells. The proteins encoded by OsCYP19-4 AS isoforms exhibited different localizations in *N. benthamiana* epidermal cells (Figure 3C). The GFP fusion of fully spliced OsCYP19-4.1-GFP was observed near the boundaries between cells in all epidermal cells, as previously reported. By contrast, OsCYP19-4.2-GFP and OsCYP19-4.3-GFP were detected solely in the guard and/or stomata subsidiary cells. As expected, the OsCYP19-4.5-GFP isoform was localized to the endoplasmic reticulum (ER) by the N-terminal ER signal peptides, and only two amino acids of the original protein remained fused to GFP after localization to the ER (Supplementary Figure S4). Because OsCYP19-4.5 should be a nonfunctional protein, it is difficult to say whether it serves any function under cold stress.

### 2.5. OsCYP19-4.2/3 AS Isoforms also Interact with AtRCN1in Guard and Subsidiary Cells

**Figure 4:**
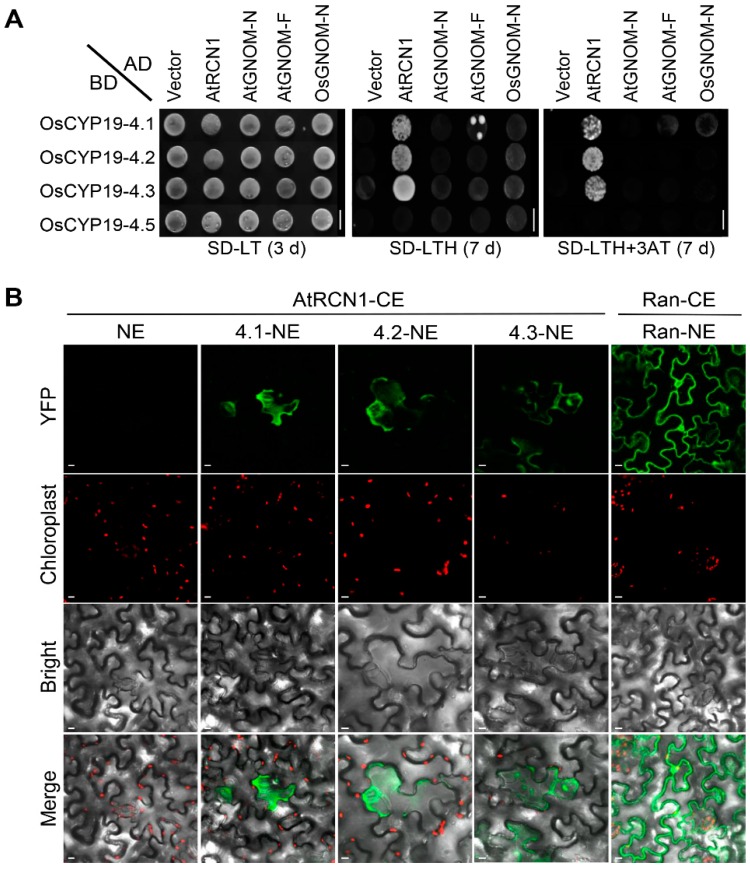
Specific protein-protein interactions between OsCYP19-4 isoforms and AtRCN1 in vivo. (**A**) Yeast two-hybrid analysis of OsCYP19-4 AS isoforms with AtRCN1, AtGNOM-N, N-term (AtGNOM-N: aa 1‒250); AtGNOM-F full length (AtGNOM-F: aa 1‒1451), and OsGNOM-N, N-term (OsGNOM-N: aa 1‒250). Specific protein-protein interactions occur between AtRCN1 and OsCYP19-4.2/3, but not OsCYP19-4.5. Scale bars = 0.7 cm; (**B**) Visualization of OsCYP19-4 AS isoforms and AtRCN1 interactions using bimolecular fluorescence complementation (BiFC). Images show YFP fluorescence signals (green color) from AtRCN1 interacting with OsCYP19-4.1 (4.1-NE), OsCYP19-4.2 (4.2-NE), or OsCYP19-4.3 (4.3-NE). Chlorophyll autofluorescence is shown in red color. All images are single confocal sections. Scale bars = 10 μm.

Our previous studies showed that OsCYP19-4 can interact with Arabidopsis PP2A (AtRCN1), but not with AtGNOM [18]. The differential protein localization observed in OsCYP19-4 isoforms might reflect a direct or indirect effect of interactions with GNOM and RCN1. Therefore we investigated the interactions between OsCYP19-4 isoform proteins and GNOM or RCN1 in Y2H and BiFC analyses. As shown in Figure 4A, yeast cells co-expressing AtGNOM-N (C-terminal deletion construct; amino acid residues 1‒250) or AtGNOM-F (full-length construct; amino acid residues 1‒1451) along with OsCYP19-4 isoforms failed to grow on selective medium (SD-LTH+3-AT). Unlike GNOM, AtRCN1 interacts physically with OsCYP19-4 isoforms, OsCYP19-4.2 and OsCYP19-4.3 as well as with OsCYP19-4.1 but not with OsCYP19-4.5 as indicated by cell growth on the selective medium. These results showed that the N-terminal 41‒90 amino acids of OsCYP19-4 are required for the interaction with AtRCN1. The AS isoforms may inhibit the interaction between OsCYP19-4.1 and AtRCN1 by forming nonfunctional heterodimers; therefore, we examined the self-interactions of OsCYP19-4 isoforms by Y2H assay. Specifically, OsCYP19-4.1 was fused to the Gal4-AD and the OsCYP19-4.2/3/5 AS isoforms were fused to the Gal4-binding domain (BD). No growth was detected on selective media, suggesting that no interactions occurred between the OsCYP19-4 isoforms (i.e., heterodimers were not formed) (Supplementary Figure S5).

Next, we performed BiFC assays to determine whether OsCYP19-4 isoforms actually interact with AtRCN1 in plant cells. To this end, split YFP fusions of OsCYP19-4 isoforms and AtRCN1 were transiently expressed in *N.benthamiana* leaves by agroinfiltration. Tobacco cells co-expressing all three complexes (CYP19-4.1‒RCN1‒YFP, CYP19-4.2‒RCN1-YFP and CYP19-4.3‒RCN1‒YFP) displayed complementation signals in both stomata guard cells and the subsidiary cells, as in our previous BiFC experiment [18] (Figure 4B). Ran-CE/Ran-NE and AtRCN1-CE/NE were used as a positive and negative controls, respectively. Expression of all the Y2H, PPIase activity and BiFC constructs was verified by immunoblot analysis (Supplementary Figure S6).

## 3. Discussion

Given that AS is a key regulatory mechanism involved in most plant biological processes, a major future challenge for plant biology is to uncover the biological significance of AS transcripts through in vivo functional analysis. However, at present, surprisingly little is known about the biochemical and physiological roles of AS transcripts in plants. In this study, we characterized eight AS transcripts of *OsCYP19-4* produced under cold stress. RT-PCR amplification and sequencing revealed that cold stress in rice seedlings caused exon skipping and intron retention in *OsCYP19-4* (Figure 1). Our data are consistent with the view that plant stress-associated genes are particularly prone to AS.

In our prediction of alternative splice sites of *OsCYP19-4*, we identified two dual-aspect splice sites that would lead to alternative splice events of introns 1 and 4 (Figure 2A). However, these AS sequences would not explain retention of introns 1, 3 and 6. Most current methods based only DNA sequence information have not achieved accurate discrimination of alternative and constitutive splicing sites largely because the conserved signals at AS junctions are similar to those of the constitutive ones [22]. Thus, our data demonstrated that DNA structural information alone may be insufficient for AS prediction.

Intron retention is the most prevalent type of AS in plants [23], and the AS events identified in this work also arose primarily from intron retentions, with the exception of *OsCYP19-4.2*; consequently, most of the isoforms we detected contained PTCs. The three different structural isoforms of OsCYP19-4 were transiently expressed from a recombinant vector and analyzed (Figure 3). Normal OsCYP19-4 protein had PPIase activity and was expressed in all epidermal cells, whereas the variants virtually lacked PPIase activity and were detected solely in guard and subsidiary cells (Figure 3). The different cellular localization between the fully spliced protein and AS isoform protein proposes that they can play a different role in a cell-specific manner. In addition, we cannot rule out the possibility that AS isoforms are only stable when they are forming a complex with active RCN1 in guard and subsidiary cells. AS transcripts containing PTCs are likely targets for NMD, or may alternatively generate truncated proteins that act as dominant negative regulators [17]. Y2H and BiFC analyses revealed no significant difference between OsCYP19-4.1 and the AS isoforms (OsCYP19-4.2/3) in the interaction with AtRCN1 (Figure 4). Given that RCN1 is a general positive regulator of ABA signal transduction in the guard cells, our results suggest that OsCYP19-4 AS isoforms play a role in stomatal physiology. Therefore, OsCYP19-4 AS isoform proteins may be involved, via their interaction with RCN1, in the mechanism of cold acclimation in guard and subsidiary cells.

We characterized cold-specific OsCYP19-4 isoforms and found that, although they had negligible PPIase activity, they could act as RCN1 interactors. In addition, to expanding our understanding of the OsCYP19-4 AS isoforms, our findings suggest that cold-specific splicing of *OsCYP19-4* plays an important role in the acclimation mechanism. Additional studies are needed to clarify the specific contribution of OsCYP19-4 AS isoforms to cold stress. Our results support the idea that *OsCYP19-4* can be used as a model for the study of multiple AS in response to abiotic stress.

## 4. Materials and Methods

### 4.1. Plant material, Growth Conditions and Cold Stress Treatments

Seeds of rice (*Oryza sativa* ssp. Japonica cv Dong-Jin, Suwon, Korea) were surface-sterilized with 70% alcohol for 5 min, treated with 50% clorox for 40 min, and washed eight times in sterilized water. These seeds were placed onto 1/2 MS media containing 0.4% (*w*/*v*) Phytagel and grown in a plant growth chamber under controlled conditions (12 h light/12 h dark at 28 °C and 75% relative humidity). To analyze the expression of *OsCYP19-4* genes, 10-day-old rice seedlings were exposed to cold by incubation in a 4 °C room. Whole seedlings were sampled after 0, 1, 3, 6, 9, 12, 24 and 48 h of cold stress, immediately frozen in liquid nitrogen, and stored at −72 °C until RNA extraction.

### 4.2. RNA Extraction and Semi-Quantitative Reverse Transcription-Polymerase Chain Reaction

Total RNA was isolated from wild-type rice seedlings at different time points using RNAiso Plus (TaKaRa, Tokyo, Japan). To remove genomic DNA, total RNA was treated with RNase-free DNase Ι (RQ1 RNase-Free DNase; Promega, Madison, WI, USA). RT Premix (SolGent, Daejeon, Korea) was used for cDNA synthesis. cDNA was subjected to semi-quantitative RT-PCR, and the density of each band was normalized against *ACTIN* transcript levels. Primer sequences are listed in Supplementary Table S1. PCR products were electrophoresed on a 1.5% agarose gel and purified using the AccuPower^®^ Gel Purification Kit (Bioneer, Daejeon, Korea). Purified PCR products were ligated with T-blunt vector at 16 °C overnight, and the resultant plasmids were used to transform *Escherichia coli* DH5α. T-blunt vector clones of *OsCYP19-4 AS* variants were sequenced using the M13-F and M13-R primers.

### 4.3. Analysis of the 5′ and 3′ Splice Site

Prediction of the 5′ (donor site) and 3′ (acceptor site) splice sites was performed using SplicePort, an Interactive Splice Site Analysis Tool (http://spliceport.cbcb.umd.edu/), ASSP (http://wangcomputing.com/assp/), and NetGene2 (http://www.cbs.dtu.dk./services/netGene2/). For calling of splice sites, the sensitivity value was set at a score threshold of ≥0.2. To identify motifs in a group of intron sequences, we used the MEME tool (http://meme.ncbr.net) [24], which returns consensus patterns or position matrices based on the results of DNA motif discovery. The resultant consensus sequences at the 5′ and 3′ splice sites and the branch point sequences of the intron‒exon splice boundaries were investigated using WebLogo, a Sequence Logo Generator (http://weblogo.berkeley.edu/logo.cgi) [25]. A logo represents each column of an alignment as a stack of letter icons corresponding to the four nucleotides; the overall height of the stack indicates the amount of information at each position, and the relative height of each letter is proportional to the observed frequency of the corresponding nucleotide.

### 4.4. Cellular Localization of OsCYP19-4 AS Isoforms

Subcellular localization assays were performed using the binary expression vector pCAMBIA1302 containing the gene encoding green fluorescent protein (GFP). To express GFP fusions of OsCYP19-4 AS isoforms cloned into the pCAMBIA1302 vector, the respective recombinant vectors and the P19 silencing suppressor plasmid were co-transformed into *Agrobacterium* strain GV3101 and co-injected into *Nicotiana benthamiana* leaves. The infiltrated leaves were monitored for GFP fluorescence in epidermal cells 2 days after agro-infiltration. Fluorescence imaging was conducted using a confocal laser scanning microscope system (LSM510 META; Zeiss, Oberkochen, Germany). RFP-BiP was co-expressed with OsCYP19-4.5-GFP in *N. benthamiana* as an ER marker.

### 4.5. Y2H Assay

The full-length CDS of OsCYP19-4 (OsCYP19-4.1) or AS forms (OsCYP19-4.2, OsCYP19-4.3, and OsCYP19-4.5) was cloned into vector pGBKT7. Full-length AtRCN1, N-terminal residues 1‒250 of AtGNOM (AtGNOM-N), full-length AtGNOM (AtGNOM-F), and N-terminal residues 1‒250 of OsGNOM (OsGNOM-N) were cloned into pGADT7. Primers used for cloning are described in Supplementary Table S1. Each of the OsCYP19-4 AS plasmid constructs with an activation domain (AD) (pGADT7) was co-transformed into yeast strain AH109 cells, and colonies were selected on leucine and tryptophan-deficient synthetic dextrose (SD-LT) agar plate at 28 °C for 7 days. Selected colonies were spotted onto agar plates containing the following media: SD-LT (synthetic dextrose [SD] lacking leucine and tryptophan), SD-LTH (SD lacking leucine, tryptophan, and histidine), or SD-LTH containing 1 mM 3-amino-1,2,4-triazole (3-AT). After plating, the cells were allowed to grow for 7 days. SD-LTH+3AT plates were used to search for protein‒protein interactions. OsCYP19-4 AS isoforms harboring the pGADT7 empty vector were used as negative controls.

### 4.6. Bimolecular Fluorescence Complementation Assaay

Interactions between AtRCN1 and OsCYP19-4 AS isoforms were investigated using the BiFC assay as previously described [18]. OsCYP19-4 AS isoform cDNAs, OsCYP19-4.1, OsCYP19-4.2, OsCYP19-4.3, and OsCYP19-4.5, were cloned into pSPYNE-35S, and *AtRCN1* cDNA was cloned into pSPYCE-35S. The BiFC constructs and P19 silencing suppressor plasmid were co-expressed in 4-week-old *Nicotiana benthamiana* leaves by *Agrobacterium tumefaciens* infection. The leaves were monitored for YFP fluorescence 48 h post-infiltration. Tobacco leaf epidermal cells were imaged using a Zeiss R510 confocal laser scanning microscope.

### 4.7. Immunoblot

Total proteins were extracted from transformed yeast cells or *Agrobacterium*-infiltrated *N. benthamiana* leaves using a crude protein extraction buffer (120 mM Tris pH 8.8, 1% SDS, 10% glycerol). The total protein extracts were separated on 12% SDS-PAGE gel and transferred onto polyvinylidene difluoride (PVDF) membranes (Thermo Fisher Scientific, Waltham, MA, USA). The membranes were probed with anti-HA (Sigma, St. Louis, MO, USA), and anti-MYC (Abcam, Cambridge, UK) antibodies overnight at 4 °C. After HRP-conjugated secondary antibody probing, HRP signals were detected using a West Pico Chemiluminescent substrate (Thermo Fisher Scientific, Waltham, MA, USA) and an MyECL Imager (Thermo Fisher Scienctific, Waltham, MA, USA).

### 4.8. In Vitro PPIase Activity Assay

Recombinant His-tagged OsCYP19-4.1 and OsCYP19-4.2 were produced using the pET28b expression vector in *E. coli*. Protein expression was induced by the addition of IPTG to the culture, and recombinant proteins were purified using an ExiProgen™ protein synthesizer (Bioneer, Daejeon, Korea) as described in Dae Hwa Yoon et al. [18]. Comparative PPIase activity analyses of recombinant OsCYP19-4.1 and OsCYP19-4.2 isoforms were performed in vitro using the tetrapeptide substrate Suc-AAPF-pNA (N-succinyl-Ala-Ala-Pro-Phe p-nitroanilide; Sigma-Aldrich, St. Louis, MO, USA) in a chymotrypsin-coupled assay, as described in our previous work [18].

## Supplementary Materials

Supplementary materials can be found at http://www.mdpi.com/1422-0067/17/7/1154/s1.
