# Supplementary material for: The OsCYP19-4 Gene Is Expressed as Multiple Alternatively Spliced Transcripts Encoding Isoforms with Distinct Cellular Localizations and PPIase Activities under Cold Stress
Source: Int J Mol Sci. 2016 Jul 19;17(7):1154. doi: 10.3390/ijms17071154 (PMC4964526; doi:10.3390/ijms17071154)
Supplement: Supplementary file 1 [file ijms-17-01154-s001.pdf]

Supplementary Materials: The OsCYP19-4 Gene Is Expressed as Multiple Alternatively Spliced Transcripts Encoding Isoforms with Distinct Cellular Localizations and PPIase Activities under Cold Stress

Areum Lee, Sang Sook Lee, Won Yong Jung, Hyun Ji Park, Bo Ra Lim, Hyun-Soon Kim, Jun Cheul Ahn and Hye Sun Cho

Table S1. List of primers forward primer (F) and reverse (R) primers used in this study.

Primer Name	Primer Sequence (5′-3′)	Application
5′UTR-OsCYP14-F	ATTTATTAGGAGGTTGCTGC	RT-PCR
OsCYP14-R SmaI	TCACCCGGGTCACTTCAGTTCGCCGCTGTCTGA	RT-PCR, Y2H assay
OsCYP14-F NcoI	ATACCATGGCGGCGAGGGAGACGT	Subcellular localization
OsCYP14.2-R SpeI	CACACTAGTGTTCGCCGCTGTCTGATA	Subcellular localization
OsCYP14.3-R SpeI	GCCACTAGTCATTGAAGAAATGTAGTATGA	Subcellular localization
OsCYP14.5-R SpeI	CACACTAGTATCGAGTACCTGGGCGAGAGA	Subcellular localization
OsCYP14-F NdeI	TACCATATGGCGGCGAGGGAGACGTC	Protein expression
OsCYP14.1-R XhoI	ATCTCGAGCTTCAGTTCGCCGCTGTCTGA	Protein expression, BiFC assay
OsCYP14.2-R XhoI	ATCTCGAGGTTCGCCGCTGTCTGATATGA	Protein expression, BiFC assay
OsCYP14-F SpeI	ATACTAGTATGGCGGCGAGGGAGACG	BiFC assay
OsCYP14.3-R XhoI	CGGCCTCGAGCATTGAAGAAATGTAG	BiFC assay
OsCYP14.5-R XhoI	TACTCGAGATCGAGTACCTGGGCGAG	BiFC assay
AtRCN1-F XbaI	ACCTCTAGAATGGCTATGGTAGATGAACCG	BiFC assay
AtRCN1-R SmaI	ATTCCCGGGGGATTGTGCTGCTGTGGAACCA	BiFC assay
OsCYP14-F EcoRI	TCAGAATTCATGGCGGCGAGGGAGACGTC	Y2H assay
OsCYP14.2-R BamHI	CAAGGATCCTCAGTTCGCCGCTGTCTGATA	Y2H assay
OsCYP14.3-R BamHI	CAAGGATCCTTACATTGAAGAAATGTAGTA	Y2H assay
OsCYP14.5-R BamHI	CAAGGATCCTCAATCGAGTACCTGGGCGAG	Y2H assay
AtRCN1-F EcoRI	ACCGAATTCATGGCTATGGTAGATGAACCG	Y2H assay
AtRCN1-R XhoI	ATTCTCGAGGAATTGTGCTGCTGTGGAACCA	Y2H assay
AtGNOM-F SmaI	CAACCCGGGAATGGGTCGCCTAAAGTTGCA	Y2H assay
AtGNOM-R(1-250) SacI	CGGGAGCTCTACTCCAGCTTTCTCTTGTTTG	Y2H assay
OsGNOM-F EcoRI	TCAGAATTCATGGGCGGCCTGAGGGCAGCG	Y2H assay
OsGNOM-R(1-250) SmaI	TCACCCGGGTTAACCTTGGTTCTTGCTACAAGC AGTCTGCCCGTCGGCAACCACGGTGGC	Y2H assay
OsGNOM-R(full) SmaI	ATTCCCGGGAACATTCACGCCTTCAGATTGTGC TGGACTATCTGACTTG	Y2H assay

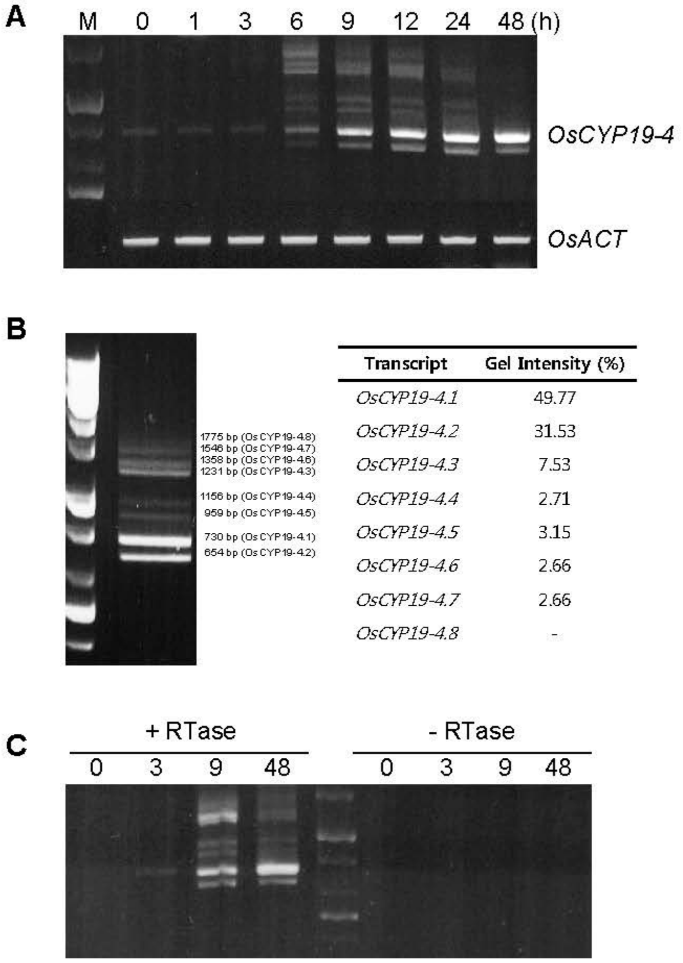

Transcript	Gel Intensity (%)
OsCYP19-4.1	49.77
OsCYP19-4.2	31.53
OsCYP19-4.3	7.53
OsCYP19-4.4	2.71
OsCYP19-4.5	3.15
OsCYP19-4.6	2.66
OsCYP19-4.7	2.66
OsCYP19-4.8	-

Figure S1. Detection of AS variants of *OsCYP19-4*. (**A**) Time course analysis of *OsCYP19-4* expression identified multiple isoforms differentially expressed after cold treatment; (**B**) Multiple PCR products for *OsCYP19-4* represent various distinct AS variants under cold stress. The intensities of the isoforms were measured by ImageJ software at the 9 h time point; (**C**) PCR reactions were performed with/without reverse transcriptase (RTase) to confirm the absence of genomic DNA contamination.

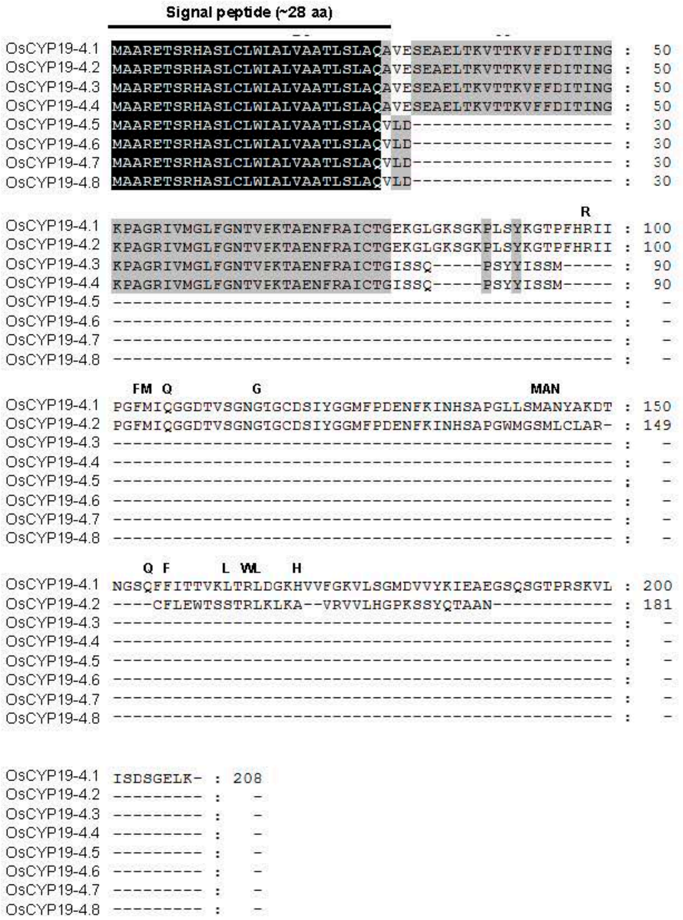

Figure S2. Polypeptides potentially encoded by AS isoforms of OsCYP19-4. Multiple sequence alignment of OsCYP19-4 AS isoforms. Amino acids necessary for CsA binding (as determined for hCYPA) are marked in bold letters. The different backgrounds indicate amino acid similarity: black, 100%; grey, 60%.

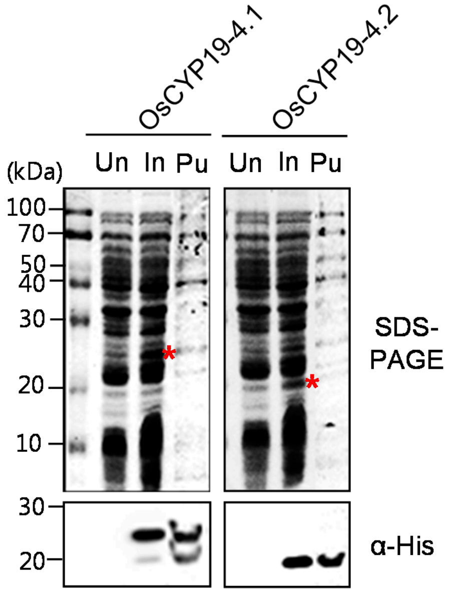

Figure S3. Expression and purification of recombinant OsCYP19-4.1 and OsCYP19-4.2 proteins in *E. coli*. Expression of OsCYP19-4.1 and OsCYP19-4.2 in *E. coli* was induced by treatment with IPTG for 2 h, and the resulting proteins were analyzed by 12% SDS-PAGE. Un, un-induced; In, induced by 1 mM IPTG for 2 h; Pu, purified protein; His, anti-His immunoblot. * indicates induced recombinant OsCYP19-4.1 and OsCYP19-4.2 proteins.

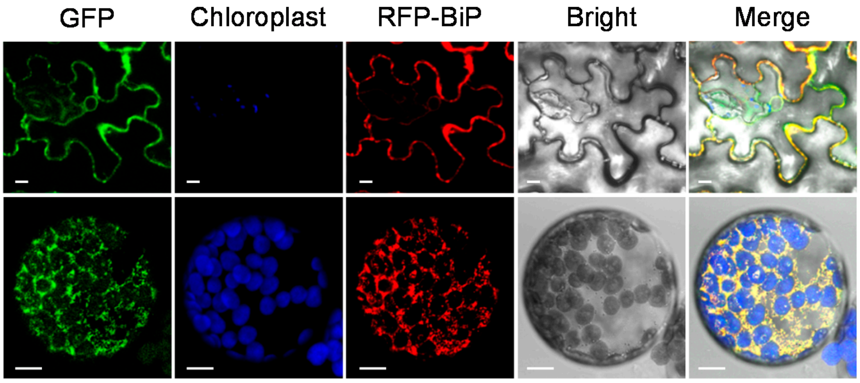

Figure S4. Co-localization of OsCYP19-4.5-GFP and RFP-BiP, used as an ER marker in *Nicotiana bent hamiana*. GFP and RFP are shown in green and red color, respectively. Scale bars = 10 μm.

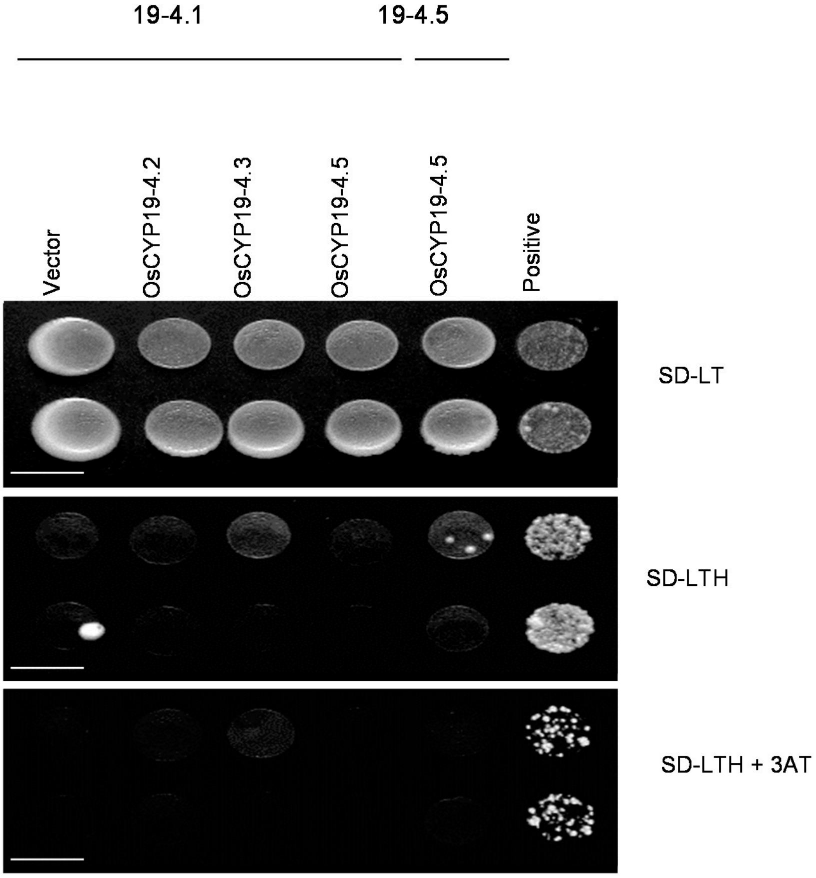

Figure S5. Analysis of interaction between OsCYP19-4 AS isoforms in *yeast*. Yeast cells expressing OsCYP19-4 AS isoforms were selected on leucine- and tryptophan-deficient synthetic dextrose (SD-LT) agar plates at 28 °C for 7 days. Selected colonies were spotted onto SD-LT, SD-LTH, and SD-LTH containing 1 mM 3-amino-1,2,4-triazole (3-AT), and grown for 7 days. No combinations grew on SD-LTH or SD-LTH+3AT selective medium. 19-4.1, OsCYP19-4.1 isoform protein; 19-4.5, OsCYP19-4.5 isoform protein. Scale bars = 0.7 cm.

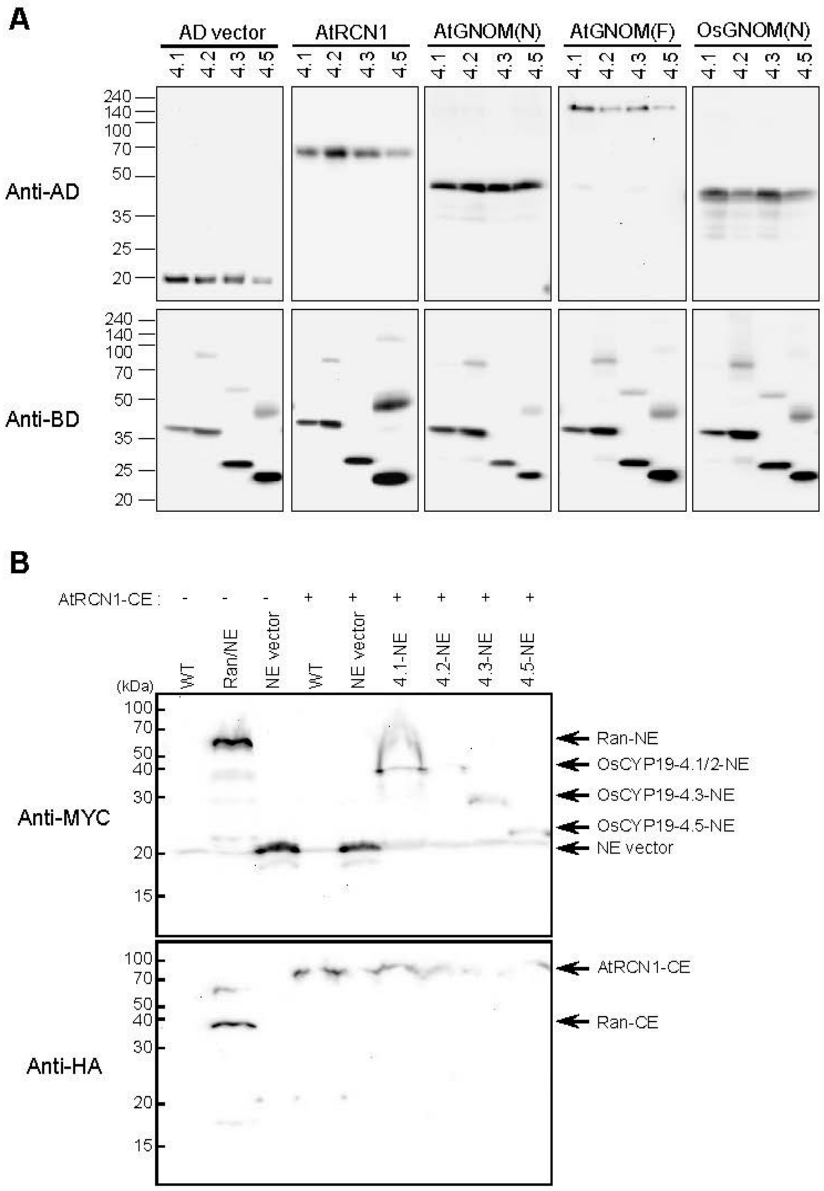

Figure S6. Immunoblot assay of Y2H and BiFC constructs. (**A**) Proteins were extracted from the indicated yeast cells grown in liquid SD-LT media. Gal4-activation domain (AD)—fused AtRCN1, AtGNOM-N, and AtGNOM-F proteins were detected with anti-AD antibody. Gal4-binding domain (BD)—fused OsCYP19-4 AS isoforms were detected with anti-BD antibody; (*B*) Proteins were extracted from *N. benthamiana* leaves after 2 days of agroinfilteration. YFP N-term (NE)—fused RAN, OsCYP19-4.1, OsCYP19-4.2, OsCYP19-4.3, and OsCYP19-4.5 were detected using anti-MYC antibody. YFP C-term (CE)—fused AtRCN1 and RAN were detected with anti-HA antibody.
